# Altered expression of the caffeine synthase gene in a naturally caffeine-free mutant of *Coffea arabica*

**DOI:** 10.1590/S1415-47572009005000090

**Published:** 2009-12-01

**Authors:** Mirian Perez Maluf, Carla Cristina da Silva, Michelle de Paula Abreu de Oliveira, Aline Gomes Tavares, Maria Bernadete Silvarolla, Oliveiro Guerreiro

**Affiliations:** 1 Embrapa Café, Brasília, DFBrazil; 2 Instituto Agronômico, Centro de Café Alcides Carvalho, Campinas, SPBrazil

**Keywords:** coffee, cup quality, differential expression, naturally decaffeinated

## Abstract

In this work, we studied the biosynthesis of caffeine by examining the expression of genes involved in this biosynthetic pathway in coffee fruits containing normal or low levels of this substance. The amplification of gene-specific transcripts during fruit development revealed that low-caffeine fruits had a lower expression of the theobromine synthase and caffeine synthase genes and also contained an extra transcript of the caffeine synthase gene. This extra transcript contained only part of exon 1 and all of exon 3. The sequence of the mutant caffeine synthase gene revealed the substitution of isoleucine for valine in the enzyme active site that probably interfered with enzymatic activity. These findings indicate that the absence of caffeine in these mutants probably resulted from a combination of transcriptional regulation and the presence of mutations in the caffeine synthase amino acid sequence.

## Introduction

*Coffea arabica*, an autogamous, allotetraploid (2n = 4x = 44 chromosomes) species that probably resulted from the natural hybridization of two diploid species, *C. canephora* and *C. eugenioides* (Lashermes *et al.*, 1999), is the most important species cultivated for coffee production, primarily because of the excellent beverage quality provided by its beans. Several parameters are associated with the high cup quality of *C. arabica*, including genetic background, environmental conditions, cultivation methods and post-harvest processing. The influence of genetic background on final cup quality is related mainly to the biochemical composition of the fruits and beans. Several studies have shown that coffee species and cultivars vary in their sugar, phenolic compound, lipid and total soluble solid content (Mazzafera *et al.*, 1998; Bradbury, 2001; Aguiar *et al.*, 2005).

Of the compounds present in coffee beans, caffeine is one of the best known and studied because of its physiological effects on humans and its role in the physiology and life cycle of coffee plants. The caffeine content of coffee beans (expressed as the percentage dry weight) varies among *Coffea* species: *C. arabica* beans contain ~1.2% caffeine, *C. canephora* 2.4% and *C. eugenioides* 0.3-0.8% (Mazzafera *et al.*, 1997), whereas the beans of *C. pseudozangebariae* and *C. richardii* do not contain caffeine (Campa *et al.*, 2005).

Caffeine biosynthesis involves several adenine-methylation steps, of which the major intermediates are methylxanthosine and theobromine [see Ashihara *et al.* (2008) for a comprehensive review of caffeine biosynthesis in coffee plants]. Three major N-methyltransferases, namely, 7-methylxanthosine synthase, theobromine synthase and caffeine synthase, are responsible for this synthesis. The genes encoding these three enzymes have been cloned from different plant species, including coffee (Ogawa *et al.*, 2001; Mizuno *et al.*, 2003; Uefuji *et al.*, 2003). These studies have identified two genes for caffeine synthase, the enzyme responsible for the final steps of caffeine synthesis. Although both enzymes share high sequence homology, they differ in their patterns of expression, with the CCS1 gene being detected in leaves and fruits (Mizuno *et al.*, 2003) and the CaDXMT1 gene mainly in fruits (Uefuji *et al.*, 2003).

In recent years, breeding programs have attempted to develop coffee cultivars with reduced levels of caffeine. However, this trait has not been successfully transferred through genetic crosses between *C. arabica* and low-caffeine species, mainly because of difficulties in interspecific crosses (Mazzafera and Carvalho, 1992). Recently, *C. canephora* transgenic plants with low levels of theobromine and caffeine in their leaves were developed based on the use of RNAi corresponding to the 3'-UTR sequences of the theobromine synthase gene (Ogita *et al.*, 2003, 2004). The results obtained so far suggest that this strategy may be a useful means of developing caffeine-free cultivars.

In addition to genetic manipulation, the natural variability in the caffeine content of wild accessions of *C. arabica* has also been evaluated (Silvarolla *et al.*, 2000). These investigations led to the identification of three coffee plants that were nearly caffeine-free in the *Coffea* Germplasm Collection of the Agronomic Institute of Campinas (IAC) in São Paulo State, Brazil (Silvarolla *et al.*, 2004). These plants were named AC1, AC2 and AC3 in honor of Dr. Alcides Carvalho. Biochemical analysis showed that the leaves of AC1 accumulated theobromine, the immediate precursor of caffeine, and that they had no caffeine synthase activity. These results were very exciting since they raised the possibility of developing naturally decaffeinated *C. arabica* cultivars. However, the development of adequate breeding strategies for the transfer of this trait to other cultivars requires adequate genetic and molecular characterization of these low-caffeine plants.

In this work, we investigated the expression profile of the three N-methyltransferase genes involved in the caffeine biosynthetic pathway. Transcripts were amplified from fruits of the AC1 mutant throughout development and maturation. In addition, the transcript amplification profile was compared with that of other *C. arabica* cultivars and with the fruits of *C. canephora* and *C. eugenioides*, the original parental species. Our results indicated that reduced expression of the caffeine synthase gene and alternative splicing were the main mechanisms for controlling gene expression during caffeine biosynthesis. We also cloned and sequenced the caffeine synthase gene from AC1 and identified nucleotide polymorphisms that may lead to changes in essential amino acids.

## Materials and Methods

###  Plant material

**Figure 1:**
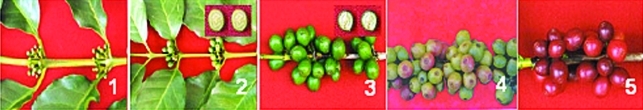
Phenological scale for coffee fruit development according to Pezzopane *et al.* (2003). 1 - pinhead, 2 - expansion, 3 - green, 4 - yellowish-green, 5 - cherry.

Fruits from low-caffeine *C. arabica* AC1 were collected during the crop year 2004/2005, based on a previously proposed phenological scale (Pezzopane *et al.*, 2003) that defined the development and maturation stages as pinhead, expansion, green, yellowish-green and cherry (Figure 1). Fruits from *C. arabica* cultivar Mundo Novo, *C. eugenioides* and *C. canephora* were also collected. The caffeine content of these accessions was 1.2%, 0.8% and 2.2%, respectively. After picking, the fruits were immediately frozen in liquid N_2_ and stored at -80 °C. All of the plants evaluated were from the *Coffea* Germplasm Collection and are cultivated at the Experimental Center of the IAC in Campinas, Brazil.

###  Data mining and identification of *C. arabica N-*metylthransferase ESTs

Gene sequences corresponding to 7-methylxanthosine synthase (CmXRS1, AB034699), theobromine synthase (CTS2, AB054841), and caffeine synthase (CCS1, AB086414; CaDXMT1, AB084125) were used for Blast searches in the Coffee Genome Database (Altschul *et al.*, 1990; Vieira *et al.*, 2006). Access to this database is restricted and a password is required (www.lge.ibi.unicamp.br/cafe). The aim of these searches was to identify possible polymorphisms for these sequences. Also, since these three enzymes shared highly conserved protein domains, clustering and sequence alignment analyses of the identified ESTs allowed the selection of truly gene-specific primers. Sequence analyses were done using CLUSTAL W (Thompson *et al.*, 1994). The primers selected were: CmXRS1, forward 5'- ATGCCCG GCTCTTTCTACAG-3' and reverse 5'-CGGGCGTCTA ATTCAACTCCT-3' (expected fragment size: 300 bp); CTS2, forward 5'-CCCGTCCAGAAGGCATATTT-3' and reverse 5'-GAGAAGGCATCATAATGGG-3' (expected fragment size: 300 bp). Primers corresponding to CCS1 included different pair combinations in which the reverse primer, 5'-CAGGATACAGGGGAATGGGATC-3', was fixed and two forward primers, F2 (5'-ATACAAGAA TTGTTGCGG-3'; expected fragment size: 833 bp) and F6 (5'-GGTCCGCCCATCAAGAAG-3'; expected fragment size: 383 bp), were used. The control housekeeping gene was actin, for which the selected primers were: forward 5'-GACCTCACAGATCACCTCAT-3' and reverse 5'-GTAGTCTCGTGGATACCAGC-3'.

###  RNA extraction and RT-PCR

Total RNA was extracted from fruits using a Trizol (Invitrogen) based protocol and treated with RNAase-free DNAase I (Qiagen). RNA was quantified by formaldehyde-agarose electrophoresis and by UV spectophotometry (difference in absorbance at 220 nm – 340 nm; Shimadzu UV spectrophotometer). Gene expression was assessed semi-quantitatively and by quantitative RT-PCR. Four hundred nanograms of RNA from each sample was used for cDNA synthesis with a commercial kit (SuperScript III First-Strand Synthesis SuperMix, Invitrogen).

Semi-quantitative RT-PCR for amplification of the *N*-methyltransferase transcripts was done using 1 μL of cDNA, 1X reaction buffer, 2 mM MgCl_2_, 2 mM dNTP, 1 pmol of each primer and 0.25 U of *Taq* polymerase. The reactions consisted of 5 min at 95 °C, followed by 30 cycles of 1 min at 95 °C, 1 min at 54 °C and 1 min at 72 °C. Actin-specific primers were used as an internal control for assessing RNA integrity and initial loading. Amplified fragments were separated by electrophoresis in 1.2% agarose gels, stained with ethidium bromide and photographed under UV light. The transcripts were analyzed based on their absence/presence and the intensity of the stained bands. At least three independent reactions were evaluated for each primer and sample.

Quantitative RT-PCR was done in an ABI7300 system (Applied Biosystems) with SYBR Green kits (Invitrogen) that included SYBR green and passive reference ROX. The reaction conditions were as described elsewhere (Iskandar *et al.*, 2004). The gene-specific primers were: CCS1 (Forward 5'-GTGCGAACAAAGGGTGCAT-3'; Reverse 5'-CGAATGAATCCTAAGAAATGTGGTAA-3'), CTS2 (Forward 5'-CCCGTCCAGAAGGCATATT T-3'; Reverse 5'-GAGAAGGCAGCATCATAATGGG-3'), CmXRS1 (Forward 5'-ATGCCCGGCTCTTTCTAC AG-3'; Reverse 5'-CGGGCGTCTAATTCAACTCCT-3'). The presence of single amplicons in the PCR products was confirmed by analyzing their dissociation curves at temperatures ranging from 60 °C to 95 °C. The RT-qPCR results were analyzed with the sequence detection software SDS version 1.3.1 (Applied Biosystems) and transcript abundance was estimated using defined threshold, baseline and Ct values (Iskandar *et al.*, 2004). Three replicates of each treatment were analyzed. The GAPDH gene was used as the endogenous control (Forward 5'-TTGAAGGGCGG TGCAAA-3'; Reverse 5'-AACATGGGTGCATCCTTGC T-3'). The developmental stage cherry from the cultivar Mundo Novo was used to normalize the results.

###  Cloning and sequencing of the caffeine synthase gene

Total DNA was extracted from AC1 plants by using a CTAB/phenol protocol (Maluf *et al.*, 2005). The caffeine synthase genomic region was amplified using the F2/R primer combination (see above). After electrophoresis in 1% agarose the amplified fragment was purified and cloned in the TA PCR2.1 vector (Invitrogen) followed by large-scale amplification in *E. coli* and purification; positive plasmids were sequenced with an ABI 3700 platform. The cDNA fragments amplified from total RNA of *C. arabica* AC1 and MN and *C. eugenioides* fruits were also cloned and sequenced as described above.

## Results

**Figure 2:**
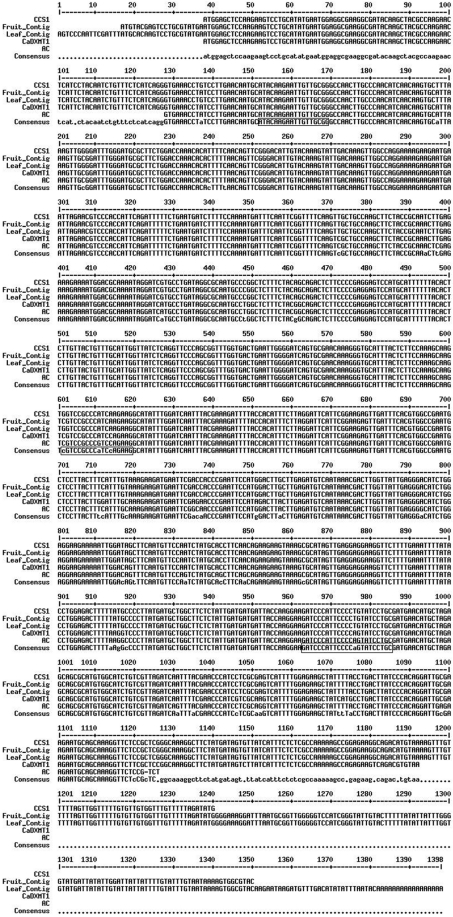
Multiple alignment of caffeine synthase cDNA sequences. Fruit and leaf contigs were assembled using ESTs identified in the Coffee Genome Database (see Material and Methods). The other sequences are CCS1 (AB086414), CaDXMT1 (AB084125.1). Boxed sequences correspond to F2, F6 and R primers.

To guarantee accurate selection of specific primers for each methyltransferase gene, the Brazilian Coffee Genome Database was searched for sequences corresponding to coffee homologs (Vieira *et al.*, 2006). Clustering and alignment analyses were done in these EST sequences until unique sequences homologous to each gene were identified. The results for these analyses are shown in Figure 2. This *in silico* analysis allowed the identification of 12 ESTs homologous only to caffeine synthase (CCS1), 11 to 7-methylxanthosine synthase (CmXRS1) and 21 to theobromine synthase (CTS2). Based on the tissues in which these ESTs were expressed and on the presence of polymorphisms in the 5'-UTR sequence, two transcripts were identified for all three genes, one in fruits and the other in leaves. The different caffeine synthase transcript sequences were (Figure 2): tissue-specific contigs resulting from the clusterization of caffeine-synthase ESTs identified in the Coffee Genome Database, cDNA amplified from fruits of AC1 and sequences deposited in GenBank. All of the sequences were highly conserved, with few nucleotide substitutions. Although previous reports indicated that the expression of CCS1 and CaDXMT1 was tissue-specific, our analysis revealed ESTs corresponding to either CCS1 or CaDXMT1 in libraries from fruits and leaves. This tissue-specific expression may be associated with polymorphism in an untranslated region (UTR). Indeed, there were differences in the 5'-UTR sequences of leaf and fruit contigs. Whether these two sequences result from alternative processing of a unique transcript or whether they represent the expression of tissue-specific alleles remains to be confirmed. In preliminary tests, the amplification of genomic regions corresponding to these three genes resulted in only one amplified fragment (data not shown). However, additional analyses are required to confirm this data. In this study, we focused on the analysis of the fruit-specific transcript.

###  Analysis of the expression of N-methyltransferases

**Figure 3:**
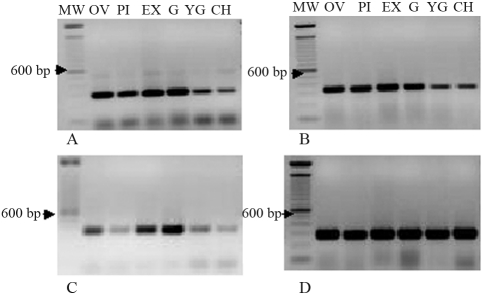
Expression of methylxanthosine synthase (A), theobromine synthase (B), caffeine synthase (C) and actin (D) in fruits of *C. arabica* cv Mundo Novo assessed by semi-quantitative RT-PCR. Fruit stages: CH – cherry, EX – expansion, G – green, OV – ovary, PI – pinhead, YG – yellowish-green.

**Figure 4:**
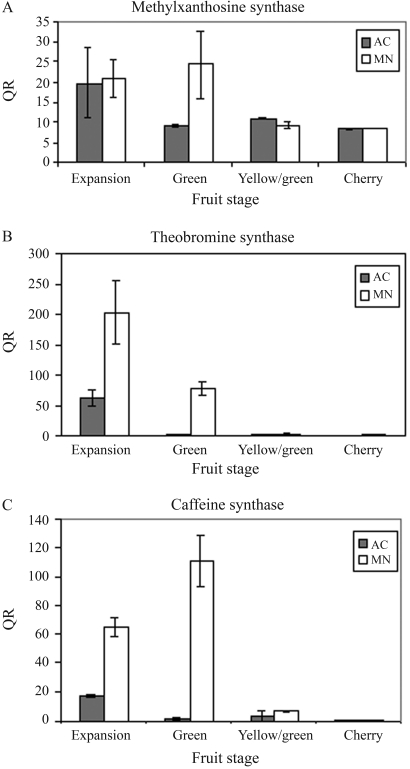
Quantification of the relative abundance of methylxanthosine synthase (CmXRS1), theobromine synthase (CTS2) and caffeine synthase (CCS1) transcripts in fruits of *C. arabica* cv Mundo Novo (MN) and AC1. Gene expression was assessed by qRT-PCR at different fruit stages.

Previous biochemical analysis of AC1 indicated an accumulation of theobromine in the leaves and fruits of this accession, and that a caffeine-degradation pathway was active in these plants (Silvarolla *et al.*, 2004). These findings suggested that the caffeine deficiency in AC1 resulted from inhibition of the biosynthetic pathway, most likely caffeine synthase activity. To confirm this, we used semi-quantitative and quantitative RT-PCR to examine the expression of the three genes involved in the biosynthesis of caffeine. Transcripts corresponding to CmXRS1, CTS2 and CCS1 were amplified in developing fruits of different *Coffea* accessions. The primers indicated in the Methods amplified unique fragments of the expected size in all of the samples (Figure 3). Quantitative analysis indicated that major differences in the expression pattern were associated either with the timing or regulation of gene expression. Transcripts of CmXRS1, CTS2 and CCS1 were present in all fruits stages of the *C. arabica* cultivar Mundo Novo, with a decline in transcript accumulation in the latter stages of fruit development (Figure 4). A similar pattern was observed for the expression of CmXRS1 in AC1 fruits (Figure 4A). In contrast, the accumulation of CTS2 and CCS1 transcripts in AC1 fruits was very low compared to that observed in Mundo Novo fruits (Figure 4B,C). For instance, CCS1 transcript levels were at least 100-fold more abundant in the green fruits of MN when compared to the same stage in AC1 (Figure 4C). In addition, CTS2 transcript accumulation in AC1 fruits declined at an earlier stage than in MN fruits. These results indicate that the regulation of these genes is altered in AC1 fruits.

**Figure 5:**
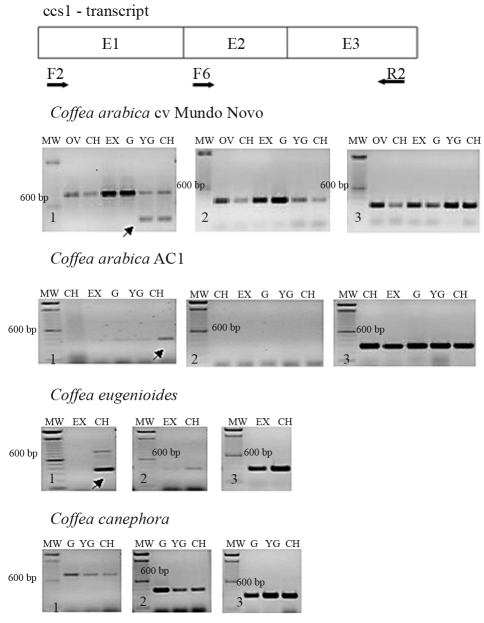
Expression of caffeine synthase (CCS1) in fruits of *Coffea* accessions assessed by RT-PCR using primer pairs (1) F2 and (2) F6. (3) Actin expression. A schematic representation of primer positions in the CCS1 transcript is shown at the top (E = exon). Fruit stages: CH – cherry, EX – expansion, G – green, OV – ovary, PI – pinhead, YG – yellowish-green. Arrows indicate the extra 360-bp transcript (see text for details).

**Figure 6:**
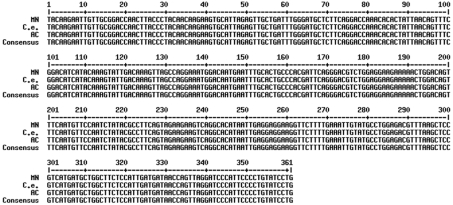
Multiple alignment of caffeine synthase extra-transcript sequences. The transcript sequences were cloned from *C. arabica* cv Mundo Novo (MN) and AC1 (AC), and *C. eugenioides* (C.e.).

**Figure 7:**
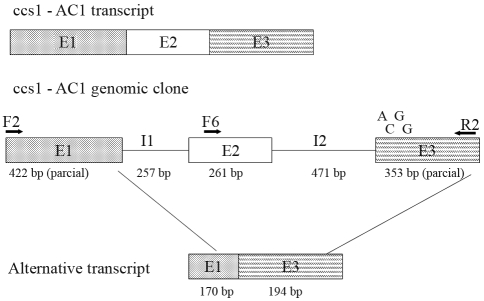
Schematic representation of the *ccs1*_AC1 sequences (corresponding to cDNA, the genomic clone and the alternative transcript). Exons (E) are represented by shaded boxes and introns (I) by lines, with their sizes indicated in base pairs (bp). The positions of the forward (F2 and F6) and reverse (R2) primers are indicated. E3 indicates the position of significant nucleotide substitutions.

Previous analysis of caffeine synthase gene expression in the leaves of AC plants indicated polymorphism in the 5'-sequence of the CCS1 transcript (Figure 2). Two forward primers (F2 and F6) that included adjacent segments of the CCS1 transcript were used to assess whether this polymorphism resulted from specific deletions in this region. Both primer combinations amplified transcripts of the expected size, *i.e.*, 830 bp for F2/R2 and 380 pb for F6/R2. However, there were significant differences in the caffeine synthase transcript profiles among the coffee plants examined (Figure 5). Transcript accumulation in Mundo Novo fruits was uniform during development, with a decrease in accumulation during the final stages. In these later stages, two fragments were amplified with the F2/R2 primer pair of the CCS1 gene: one corresponding to the expected size (800 bp) and a smaller fragment (≈360 bp). This unexpected transcript was not seen in other fruit stages or among the amplified products of other primer combinations (data not shown). Sequencing of this transcript indicated that it represented a truncated *ccs1* transcript (Figures 6  and 7).

In AC1 fruits, the alternative 360 bp transcript from the F2/R2 primer pair was preferentially amplified (Figure 5). The larger transcript was weakly amplified and only observed in one repetition, indicating that it was a rare transcript. In addition, no fragments were amplified with the F6 primer pair, indicating the occurrence of sequence polymorphism in this region (Figure 5).

Figure 5 also shows the expression of CCS1 in the parental species *C. canephora* and *C. eugenioides*. A steady, uniform amplification of CCS1 transcripts was observed in *C. canephora* fruits with both primer pairs. Expression pattern in *C. eugenioides* fruits was similar to that observed in AC1 fruits. In this case, two transcripts were also amplified, with the small one being significantly more abundant than the larger one. The F6 primer pair produced only weak amplification.

###  Polymorphism in the caffeine synthase gene

**Figure 8:**
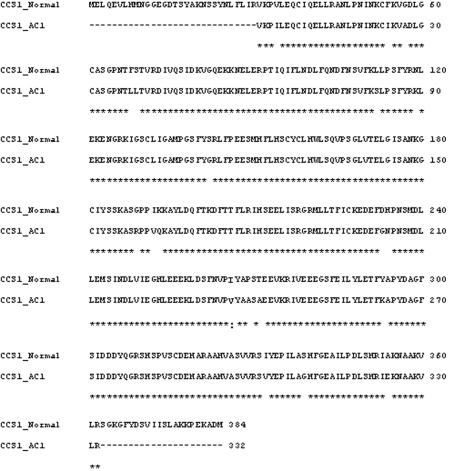
Alignment of *C. arabica* caffeine synthase protein sequences. The putative sequences correspond to the normal *ccs1* clone (AB086414) and AC1 *ccs1* clone. Double dots indicate the amino acid substitution in the enzyme active site.

To confirm transcript identity, the RT-PCR amplified fragments were cloned and sequenced, and the genomic sequence corresponding to the CCS1 coding region of AC1 was also characterized. The CCS1 gene consisted of three exons and two introns, with a total length of ~1780 bp (Figure 7). Comparison with the previously cloned CCS1, CaDXMT1 and AC1 gene sequences indicated the presence of several single nucleotide polymorphisms (SNPs; data not shown). Analysis of the putative protein sequence indicated that these SNPs lead to synonymous and non-synonymous amino acid substitutions (Figure 8). The two main polymorphisms included one associated with the F6 primer region that involved a G to C substitution, and another involving a key amino acid in exon 3 (the substitution of isoleucine at position 266 in CCS1 for valine in AC1) (McCarthy and McCarthy, 2007).

Sequencing of the 360 bp extra transcript revealed that it was a truncated transcript containing all of exon 3, part of exon 1 and none of exon 2 (Figure 7). The same sequence was observed in *C. arabica* Mundo Novo and AC1 and in *C. eugenioides*, indicating that this transcript is widely expressed in ripening fruits. Sequence comparisons between the genomic clone CCS1 and this extra transcript revealed several nucleotide polymorphisms, which suggested different genomic origins for these sequences.

## Discussion

The sequences for all methyltransferase genes involved in the biosynthesis of caffeine have previously been identified in coffee and other plant species (reviewed by Ashihara *et al.*, 2008). However, the fact that these enzymes share highly conserved protein domains makes the identification of gene-specific sequences for each methyltransferase very difficult. *In silico* analyses of previously described primers for coffee methyltransferases (Koshiro *et al.*, 2006) indicated that these primers were not completely specific for the designated gene and that they could actually anneal to more than one methyltransferase gene sequence. Consequently, expression analysis by RT-PCR may be compromised. In this study, the large number of coffee sequences available in the Brazilian EST Coffee Project meant that it was possible to accurately select gene-specific primers. In addition, all of the primers used here were selected from a conserved consensus region of each gene, which meant that the expression of all possible alleles was assessed.

Caffeine is one of the best studied compounds in coffee beans, and the characterization of its metabolic pathway is of great importance for future breeding strategies. In a recent study (Koshiro *et al.*, 2006), the expression of CmXRS1, CTS2 and CCS1 was evaluated in developing fruits from the *C. arabica* cultivar Mokka and *C. canephora*. These authors reported regular expression of CmXRS1 and CCS1 during fruit development, with a decrease in accumulation in later stages, as also observed here in *C. arabica* Mundo Novo fruits. In contrast, they observed irregular expression of CTS2 during fruit development, whereas we observed peaks of CTS2 transcript accumulation in the expansion stage (Figure 4B). In AC1 fruits, CTS2 transcript accumulation declined at earlier stages (Figure 4B) and CCS1 transcript levels were strongly reduced in all fruit stages (Figure 4C). These differences in CTS2 transcript accumulation may be related to mechanisms of feedback regulation. The accumulation of theobromine, a product of CTS2 activity and substrate for CCS1, in AC1 fruits may activate a cellular signaling pathway to repress CTS2 transcription.

The differences in the CCS1 transcript profiles seen here during coffee fruit development may be related to mechanisms controlling gene expression, such as mutations in the promoter region, post-transcriptional events and mRNA stability. However, the analysis of caffeine synthase gene expression in AC1 and normal coffee fruits indicated an alternative hypothesis to explain caffeine biosynthesis. In *C. arabica* fruits, the synthesis of caffeine occurs in green, undeveloped fruits. In the latter stages, when the fruits are partially (yellowish-green) or fully (red) ripe, there is no caffeine synthase enzyme activity, although the caffeine content remains stable (Suzuki and Waller, 1984; Koshiro *et al.*, 2006)

The mean caffeine content of *C. arabica* fruits (~1.2%) is intermediate to that of *C. canephora* (2.4%) and *C. eugenioides* (0.8%). RT-PCR analysis indicated the accumulation of transcripts corresponding to caffeine synthase in all of the fruit stages examined. However, plants with normal caffeine synthesis amplified an extra transcript in the final stages of fruit development; *C. eugenioides*, which has a lower caffeine content than *C. arabica* and *C. canephora*, also amplified this extra transcript in cherry fruits. In contrast, AC1 fruits contained only the smaller transcript. Since AC plants have no significant caffeine synthesis or caffeine accumulation during fruit ripening (Paulo Mazzafera, personnal communication), and since neither Mundo Novo nor *C. eugenioides* plants synthesize caffeine in the latter stages of fruit development, the presence of the extra transcript may be associated with the inhibition of caffeine biosynthesis. The mechanisms involved in this inhibition remain to be investigated, but may involve the suppression of caffeine synthase expression or inhibition of enzymatic activity.

Sequence analysis of this extra transcript suggested that it may be encoded by an alternative caffeine synthase allele that was not identified in the cloning experiments. Southern blot analysis using this transcript as a probe was inconclusive (data not shown). Since all of the methyltransferases shared a highly conserved nucleotide sequence, several fragments were identified in the genotypes that were examined. We are currently developing more specific gene probes to identify and characterize this allele, and to determine whether an active protein is translated from this transcript.

In addition to their differential regulation of gene expression, AC1 mutants also contained an altered caffeine synthase gene. Analysis of the putative protein sequence indicated several amino acid substitutions, most of which may not affect enzyme activity since they potentially will not modify the enzyme structure (Andrew McCarthy, personal communication). However, structural analysis of caffeine synthase has shown that an amino acid substitution at position 266 may be relevant since this residue is crucial for substrate selection between theobromine and 7-methylxanthine (McCarthy and McCarthy, 2007). This substitution may modify the size of the active site such that theobromine will no longer fit properly. In this case, the substitution of isoleucine by valine in the AC1 gene sequence will likely affect enzyme activity (Andrew McCarthy, personal communication). Interestingly, the putative protein sequences of theobromine synthase from *C. canephora* and *C. arabica* contain valine at this position (McCarthy and McCarthy, 2007).

In conclusion, the results described here suggest that the reduced caffeine synthesis seen in AC1 may involve two distinct mechanisms, namely, the regulation of gene transcription and the modulation of enzyme activity. We are currently investigating the importance of these two possibilities. These results also provide a molecular basis for understanding AC mutants and for establishing strategies for breeding this trait into available coffee cultivars.
